# A Qualitative Study on Mothers' Experiences of Breastfeeding Cessation in Turkey

**DOI:** 10.1111/jjns.70069

**Published:** 2026-07-30

**Authors:** Seçil Hur, Ayça Solt Kirca, Nihal Avcı Başboğa

**Affiliations:** ^1^ Department of Midwifery Kırklareli University, School of Health Sciences Kırklareli Turkey; ^2^ Department of Women's Health and Gynaecological Nursing Istanbul University‐Cerrahpaşa, Florence Nightingale Faculty of Nursing Istanbul Turkey

**Keywords:** breastfeeding cessation, infant, maternal experience, qualitative research

## Abstract

**Problem/Background:**

Ending the breastfeeding process is a challenging decision for both mother and baby. However, cessation of breastfeeding is an inevitable stage for many mother‐infant dyads and often represents the baby's first experience of separation from the mother.

**Aim:**

This study aimed to explore the experiences of mothers with infants aged 0–36 months who had undergone the process of breastfeeding cessation.

**Method:**

A phenomenological qualitative research design was adopted. Participants were selected through purposive sampling from various regions of Turkey, and data collection continued until saturation (*N* = 14 mothers; mean age 29.4 years, all with infants aged 0–36 months). Semistructured online interviews were conducted. Data were coded independently by two researchers and analyzed through content analysis.

**Results:**

Thematic analysis revealed five main themes: (1) reasons for breastfeeding, (2) factors influencing the decision to cease breastfeeding, (3) methods used for cessation, (4) facilitators and barriers during the process, and (5) emotional responses to cessation.

**Conclusion:**

Mothers' decisions to cease breastfeeding were primarily influenced by fatigue, sleep deprivation, and perceived decreases in milk supply. Key facilitating factors included maternal determination, a supportive environment, and reduced breastfeeding demand from the infant. While some mothers felt relieved post‐cessation, others, particularly those who used traditional aversive methods, expressed regret.

## 1 Introduction

Breast milk is widely recognized as the optimal source of infant nutrition, providing both physiological and psychosocial benefits during the early months of life. The World Health Organization (WHO) and the United Nations Children's Fund (UNICEF) recommend exclusive breastfeeding for the first six months, followed by continued breastfeeding with complementary foods for at least two years or longer (World Health Organization 2022; UNICEF 2022). Despite its well‐documented advantages, many mothers face challenges in sustaining breastfeeding, and decisions regarding when and how to cease breastfeeding vary considerably across cultural and social contexts (Gökçay and Baslo 2002; Dinç et al. 2015; Alsaç and Polat 2018). While difficulties in initiating or maintaining breastfeeding during the early postpartum period have been widely discussed in the literature, the challenges faced by mothers who breastfeed for extended periods and later attempt to cease breastfeeding are qualitatively different. This study focuses specifically on breastfeeding cessation‐related experiences among mothers who had already sustained breastfeeding beyond the early months, rather than on early breastfeeding discontinuation.

Reasons for breastfeeding cessation are multifactorial. Commonly reported influences include perceptions of insufficient milk supply, maternal fatigue, return to work, subsequent pregnancy, and cultural practices (Altunel and Özaydın 2022; Odom et al. 2013; Denzin and Lincoln 2000). Recent studies highlight that while gradual and child‐centered breastfeeding cessation is associated with smoother maternal and infant adjustment, aversive or abrupt methods remain prevalent in many societies, often transmitted through intergenerational advice rather than professional guidance (Duffy 1985; Denzin et al. 2006; Gökdemirel et al. 2013; Ericson and Palmér 2020). Mothers who adopt these practices may experience guilt, regret, or emotional distress, while infants may display irritability or clinginess (Şolt‐Kırca and Hür 2022; Güleroğlu et al. 2023; Oflu 2020)

Although international literature on breastfeeding cessation practices has expanded in recent years, qualitative evidence focusing on maternal lived experiences remains limited. Studies from Jordan, Nigeria, and Europe emphasize that emotional, cultural, and health‐related factors intersect in shaping breastfeeding cessation decisions, yet few have explored this issue in the Turkish context (Gürarslan Baş et al. 2018; Kartal and Deniz‐Acar 2022; Hacettepe University Institute of Population Studies 2019). Understanding mothers' perspectives is essential to designing supportive, evidence‐based interventions that promote both maternal well‐being and healthy infant development.

Since the experience of breastfeeding cessation is a process that can be shaped by personal, emotional, and cultural factors, a qualitative method was deemed most appropriate for the design of this study. Specifically, a phenomenological approach allows for an in‐depth examination of mothers' experiences, meanings, and perceptions regarding the breastfeeding cessation process. This approach provides a more nuanced understanding of how Turkish mothers interpret and manage breastfeeding cessation within the context of cultural traditions, family influences, and limited professional guidance. Focusing on Turkish mothers offers valuable insights into culturally unique practices and unmet support needs that are often underrepresented in the international literature.

## 2 Research Aim

This study aimed to explore the lived experiences of Turkish mothers with children aged 0–36 months during the process of breastfeeding cessation. Specifically, it sought to identify the factors influencing cessation decisions, the methods used, the emotional and behavioral responses of mothers and infants, and the facilitators and barriers encountered throughout the breastfeeding cessation process.

Within this overall aim, the following research questions were addressed:

1. What are the breastfeeding experiences of mothers?
2. What factors influence mothers' decisions to cease breastfeeding?
3. What are the emotional and behavioral reactions of mothers and infants following cessation?
4. What are the facilitating and hindering factors during the breastfeeding cessation process?
5. What methods are used to cease breastfeeding and what are the underlying reasons for these choices?

## 3 Methods

### 3.1 Study Design

This study employed a qualitative research design using a phenomenological approach. Phenomenology seeks to understand the essence of participants' lived experiences and to identify shared patterns or themes from their perspectives^8^.

### 3.2 Setting and Duration

The study was conducted between April 2022 and June 2023 through social media platforms (Instagram, Facebook, Twitter, and WhatsApp) targeting mothers with children aged 0–36 months. A recruitment post describing the study was shared on these platforms, inviting eligible individuals to participate. Mothers who met the inclusion criteria contacted the researchers voluntarily. Data were collected via telephone interviews.

### 3.3 Sampling and Participants

A purposive sampling technique was used to identify participants who had experienced breastfeeding cessation. Data collection was concluded once saturation was achieved that is, when no new information emerged from the interviews (Duffy 1985; Denzin et al. 2006). The final sample included 14 mothers who had ceased breastfeeding their children between the ages of 0–36 months.

Inclusion criteria were; mothers aged ≥ 18 years who were primiparous or multiparous; had a child aged 0–36 months; had breastfed and subsequently experienced breastfeeding cessation during this period; voluntarily agreed to participate; had no diagnosed psychiatric illness or ongoing psychiatric treatment; had no communication or language difficulties; were able to complete all interview questions; and could participate in an online or telephone‐based interview. For mothers with more than one breastfeeding cessation experience, data were collected regarding the most recent experience.

*Exclusion criteria;* included mothers who were under 18 years of age; had a child older than 36 months; had a twin or multiple birth; had not experienced breastfeeding cessation; were unwilling or unable to participate in remote interviews; did not complete the interview; or reported psychiatric, psychological, or communication‐related problems

Information regarding breastfeeding practices was collected to contextualize mothers' experiences; however, participants were not categorized according to exclusive or mixed feeding, as the analysis focused on experiences related to breastfeeding cessation following sustained breastfeeding.

### 3.4 Sample Size and Data Saturation

The sample size was determined based on the principles of qualitative research, where depth of understanding rather than numerical representation is prioritized. Data collection continued until data saturation was achieved, defined as the point at which no new codes, themes, or insights emerged from successive interviews. In this study, saturation was reached by the twelfth interview; two additional interviews were conducted to confirm saturation, resulting in a final sample of 14 participants.

### 3.5 Data Collection

A comprehensive literature review informed the development of a semi‐structured interview guide (Alsaç and Polat 2018; Denzin et al. 2006; Gökdemirel et al. 2013). The interview form included questions on participants' breastfeeding practices, breastfeeding cessation experiences, methods used for cessation, and emotional and behavioral responses observed in both mother and child. Demographic and obstetric information was also gathered

All interviews were conducted by a single researcher who is an experienced midwife and academic. The researcher had no prior relationship with the participants. Transcription and translation were conducted by a bilingual researcher who was not involved in data collection. A second bilingual researcher independently back‐translated selected excerpts to ensure accuracy.

Participants were contacted and informed about the study prior to the interview. Verbal informed consent was obtained before beginning each session. Interviews were conducted in Turkish and audio‐recorded with the participant's permission. All interviews lasted between 18 and 22 min and were transcribed verbatim within 24 h.

To ensure data accuracy, key points were summarized and confirmed with participants during the interview. The questions were designed to be clear, concise, and free of bias, encouraging participants to share their experiences openly. No time limits were imposed, allowing each interview to continue until the topic was thoroughly explored

All interviews were conducted in Turkish. Transcripts were translated into English by a bilingual researcher with expertise in qualitative research. To ensure accuracy, another bilingual researcher performed a back‐translation of selected excerpts, and discrepancies were resolved through discussion until consensus was reached.

### 3.6 Interview Process and Data Management

Sample questions from the semi‐structured interview guide are provided in File S1. The guide included open‐ended questions, and flexible probes were used as needed to encourage clarification and deeper exploration of participants' experiences. The interview guide remained largely stable throughout the study, with minor refinements made after the pilot interview to improve clarity.

No formal field notes were taken; however, the interviewer documented key observations immediately following each interview. To build rapport and trust during telephone interviews, participants were contacted in advance, the study purpose was clearly explained, and a conversational interviewing style was adopted.

All interviews were audio‐recorded with consent, transcribed verbatim, and anonymized by removing identifiable information. Transcripts were stored securely on password‐protected devices accessible only to the research team. An audit trail was maintained through systematic documentation of coding decisions and theme development. Discrepancies during analysis were resolved through discussion between researchers until consensus was reached.

### 3.7 Researcher Positionality and Reflexivity

The interviews were conducted by the lead researcher, a midwife and academic with experience in maternal and child health and qualitative research. This professional background supported effective communication with participants while maintaining awareness of the potential influence of disciplinary perspectives on data collection and interpretation. There was no prior relationship between the researcher and the participants.

To reduce potential bias, the interview guide was pilot‐tested, and data analysis was independently conducted by two researchers. Discrepancies were resolved through discussion, and regular peer discussions during analysis enhanced reflexive consideration and credibility.

### 3.8 Data Analysis

Descriptive data obtained from the participant information forms were summarized using numerical statistics. Qualitative data were analyzed using content analysis. Audio‐recorded interviews were transcribed verbatim into Microsoft Word documents to create the raw data corpus. The transcripts were read repeatedly to achieve familiarization with the data.

Two researchers independently conducted line‐by‐line coding of the transcripts. Initial codes were compared, and an agreement level of 80% was achieved. Discrepancies were discussed through an iterative, consensus‐based process until full agreement was reached. Cohen's Kappa was not formally calculated due to the small sample size and the qualitative, consensus‐oriented analytic approach; however, the achieved level of agreement indicates substantial reliability.

Following initial coding, an iterative analytic process was undertaken. Codes were reviewed and clustered based on conceptual similarity, leading to the development of subthemes. Subthemes were further refined and organized into overarching themes through constant comparison across transcripts, considering similarities, differences, and relationships among codes. The analysis was guided by the five main interview questions to ensure alignment with the study aims.

Analytic decisions were documented throughout the process to enhance transparency and methodological rigor. Data management and coding were supported using MAXQDA 2024 software.

### 3.9 Ethical Considerations

The study received ethical approval from the Non‐Interventional Research Ethics Committee of Kırklareli University Institute of Health Sciences (Date: 28/12/2021, Decision No: 10). Participants were provided with verbal explanations regarding the study's aims, procedures, and confidentiality measures. Verbal consent was obtained before interviews. Personal identifiers were anonymized and replaced with codes such as P1, P2, … P14. Any names or locations mentioned during the interviews were pseudonymized to protect participants' privacy.

### 3.10 Reporting Guideline Statement

This study was conducted and reported in accordance with the Consolidated Criteria for Reporting Qualitative Research (COREQ) checklist. A completed COREQ checklist has been submitted as a Supporting Information file.

## 4 Results

Fourteen mothers who had ceased breastfeeding their children under 36 months of age participated in the study. The participants' average age was 31.78 ± 4.02 years (min: 26, max: 37), and 11 of them held a university degree. The mean breastfeeding duration among the participants was 23.9 months. Eight mothers reported using traditional aversive methods to cease breastfeeding, while six used communicative strategies with their infants. Detailed sociodemographic characteristics and breastfeeding cessation practices are presented in Table 1.

**TABLE 1:** Sociodemographic characteristics and breastfeeding cessation practices of the participants.

Participant	Age	Education level	Employment status	Income level^d^	Number of children^e^	Breastfeeding duration (months)^a^	Support for breasfeeding termination received^b^	Cessation method^c^
P1	28	Secondary school	Not employed	Income<expenses	3	14	Yes/nurse	Verbal communication
P2	26	University	Not employed	Income = expenses	1	24	Yes/family	Bitter paste, vinegar
P3	32	University	Not employed	Income = expenses	1	20	No	Bitter paste, vinegar
P4	35	University	Employed	Income>expenses	2	24	No	Pepper paste
P5	26	University	Not employed	Income = expenses	1	30	No	Verbal communication
P6	39	University	Employed	Income<expenses	1	32	Yes/psychologist	Verbal + chili powder, turmeric
P7	34	University	Employed	Income<expenses	1	17	No	Verbal communication
P8	29	University	Not employed	Income<expenses	1	23	No	Verbal communication
P9	31	University	Employed	Income<expenses	1	27	No	Verbal communication
P10	37	Postgraduate	Not employed	Income = expenses	2	27	No	Verbal communication
P11	36	University	Not employed	Income<expenses	2	29	No	Sudden cessation
P12	27	Postgraduate	Not employed	Income = expenses	2	22	Yes/psychologist	Covered with bandage/wrap
P13	32	University	Employed	Income>expenses	1	22	Yes/online training	Bitter paste, vinegar
P14	33	University	Employed	Income = expenses	1	24	No	Bitter paste, vinegar

^a^ Duration of breastfeeding last child (months).

^b^ Support for breastfeeding termination received.

^c^ Breastfeeding termination method.

^d^ Expenses were categorized as low, moderate, or high based on participants' self‐reported monthly household expenditures.

^e^ Number of children” refers to the total number of children per mother. At the time of the study, all participants were breastfeeding a single child.

Content analysis revealed five main themes: (1) Reasons for breastfeeding, (2) Factors influencing the decision to cease breastfeeding, (3) Breastfeeding cessation methods, (4) Facilitators and barriers in the cessation process, and (5) Emotional and behavioral reactions to breastfeeding cessation. These themes and their associated subthemes are presented in Table 2.

**TABLE 2:** Main themes and subthemes derived from content analysis.

Main themes	Subthemes
Reasons for breastfeeding	Infant's need, enjoying breastfeeding, bonding, equal treatment among children, religious reasons, education and information, healthcare professional advice
Factors influencing cessation decision	Fatigue, frustration, sleep deprivation, decreased milk supply, pregnancy, maternal health, social pressure, perceived infant satiation, infant's dependence on breastfeeding, reduced infant demand, daycare enrollment, healthcare advice
Cessation methods	Distraction, verbal reasoning, aversive methods (e.g., bitter substances)
Facilitators and barriers	Determination, maternal readiness, supportive environment, infant's understanding, reduced suckling demand, lack of support, reluctance to upset child, infant nursing to sleep
Reactions to cessation	Relief, better sleep quality, positive emotions, increased bonding, easier adaptation, improved appetite, breast engorgement, sadness, regret, stress, continued nursing demand, avoidance behavior, distress, irritability, replacement behaviors

### 4.1 Reasons for Breastfeeding

Mothers stated that they continued breastfeeding because they believed it was necessary for their infants, found it enjoyable, felt it strengthened the mother‐infant bond, aimed to treat all children equally, or were motivated by religious reasons. Other supporting factors included receiving education or information about breastfeeding and recommendations from healthcare professionals. The most cited reason was the perceived nutritional and emotional need of the infant.

Breastfeeding is definitely the child's right. I thought of it as part of the environment he was used to after leaving the womb. That's why I never offered formula… I was determined to succeed at it. (P9)

I breastfed for one and a half years with joy. Then I wanted her to stop. But since my other daughter nursed until 2.5 years, I thought this one should too. (P11)

These statements illustrate how breastfeeding was framed not only as a nutritional practice but also as a moral and relational responsibility embedded in maternal identity.

My pressure to continue until two years came mainly from religious teachings. There is a verse in the Qur'an that recommends breastfeeding until two years. But I think I pushed too hard those last two months… (P12)

This reflects how religious beliefs shaped not only the duration of breastfeeding but also the internal pressure mothers placed on themselves.

### 4.2 Factors Influencing the Decision to Cease Breastfeeding

The primary factors influencing mothers' decision to cease breastfeeding were fatigue, exhaustion, and sleep deprivation. Other factors included a perceived decline in milk supply, becoming pregnant again, maternal health concerns, social pressure, and the belief that the infant was sufficiently nourished.

It's such a tiring process. Near the end, you just want it to be over… you want to get past that point and be done with it. (P2)

These accounts suggest that cessation often emerged from cumulative emotional and physical depletion rather than a single triggering factor.

He wanted to nurse to relax or fall asleep… not for nutrition. That was overwhelming for me… I feel relieved now. I know he got what he needed, so I'm at peace. (P7)

Some mothers also noted that their infants were either too attached to the breast or showed decreasing interest in nursing, or had started daycare prompting cessation.

He became addicted to breastfeeding. He nursed all day and night like a pacifier… I couldn't even create a routine. (P5)

It was more of a comfort thing. He nursed after I came home from work, like a reunion ritual. (P7)

### 4.3 Breastfeeding Cessation Methods

Participants reported using distraction, verbal reasoning, or aversive techniques to wean their children. The most frequently reported method was aversive breastfeeding cessation using unpleasant tastes or sensations.

Friends recommended using sabır taşı (a bitter paste). I applied it and said, ‘The milk tastes bad now.’ He tasted it and never asked again. (P3)

I used the traditional method applied pepper paste. (P4)

The use of such traditional methods indicates the influence of culturally transmitted practices in shaping cessation strategies.

My mom kept insisting: ‘Put on pepper, charcoal, a smelly thing, bandage it!’ But I tried to do it sweetly and gently. I took him outside, distracted him, didn't want it to be a traumatic memory. (P9)

### 4.4 Facilitators and Barriers in the Cessation Process

Mothers stated that being determined, feeling ready, having a supportive environment, and noticing reduced interest from the child facilitated the breastfeeding cessation process.

I didn't feel sad. It was something that had to happen. I knew it wouldn't last forever. (P1)

My mom and sister told me not to worry about trauma if I stopped suddenly. That helped me relax. (P14)

Conversely, the lack of support, feeling unready, reluctance to upset the child, and the infant's habit of falling asleep while nursing were perceived as challenges.

He wasn't ready. If I offered now, he'd still nurse. He started clinging more afterward… like we had separated. (P4)

He didn't know how to sleep without nursing. His father had to step in. We're still cuddling to sleep. I wish I hadn't linked sleep to nursing. (P9)

### 4.5 Reactions to Breastfeeding Cessation

Mothers reported positive reactions such as relief, improved sleep quality, emotional well‐being, increased closeness and physical affection from their children, smoother adaptation, and increased consumption of solid foods.

No regrets. I'm so glad I did it this way… I feel no guilt. (P4)

My baby used to hate cuddling. Now he comes to hug and kiss me constantly. (P8)

I breastfed for five years straight pregnancy and nursing. I was exhausted. I felt so happy and relieved when it ended. (P10)

Some mothers, however, experienced sadness, regret, stress, breast engorgement, and discomfort. They also observed signs of distress in their children, such as irritability, persistent requests to nurse, breast avoidance, and seeking alternative comfort behaviors.

You feel like something is missing from you. (P1)

I was very emotional and cried a lot. I feared our bond would break. (P5)

Sometimes he still wants to kiss my chest… It's like his safe space. I let him, but I feel uncomfortable now. (P13)

**FIGURE 1:**
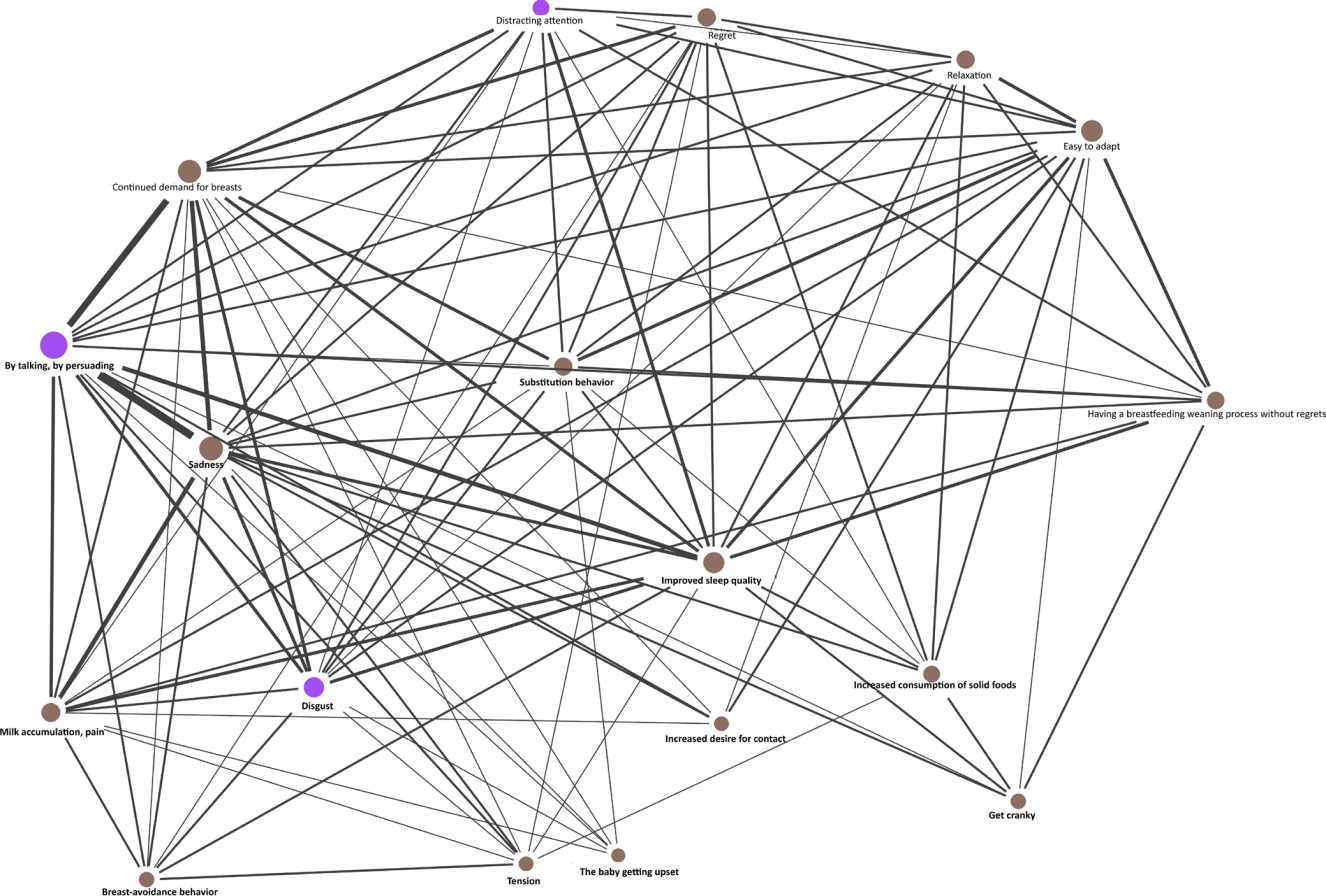
Code map. A code map illustrating the co‐occurrence of breastfeeding cessation methods and emotional responses is provided in Figure 1.

A code map illustrating the co‐occurrence of breastfeeding cessation methods and emotional responses is provided in Figure 1.

## 5 Discussion

Breastfeeding is a biologically and emotionally significant process based on the mother's anatomical and physiological abilities and the newborn's innate reflexes. It also represents a culturally shaped experience that strengthens the bond between mother and baby (Şolt‐Kırca and Hür 2022). However, a review of the literature reveals that while the initiation and maintenance of breastfeeding are largely focused on, the cessation of breastfeeding is not adequately addressed from both the mother's and the baby's perspectives (Güleroğlu et al. 2023; Oflu 2020). Cessing breastfeeding doesn't simply mean the baby stops receiving breast milk; it also represents a transitional period in which the relationship between mother and baby is restructured. Therefore, understanding how this process ends, how it is experienced, how the mother manages it, how the transition to this new phase in the mother‐baby relationship occurs, and how the cultural context influences this process is crucial.

The mean breastfeeding duration among participants was 23.9 months, which is relatively high compared to national data. Previous studies in Turkey have reported average breastfeeding durations ranging from 15.3 to 20.3 months (Altunel and Özaydın 2022; Oflu 2020; Gürarslan Baş et al. 2018; Kartal and Deniz‐Acar 2022). According to the 2018 Turkey Demographic and Health Survey, the median breastfeeding duration for children aged 0–35 months was 16.7 months (Hacettepe University Institute of Population Studies 2019). Based on the literature, it can be hypothesized that the longer breastfeeding duration observed in this study may be related to the participants' higher education levels, leading to greater knowledge and awareness of the benefits of breastfeeding. However, it is also known that higher education levels can lead to mothers' more active participation in the workforce and earlier return to work, and that increased income levels can facilitate access to formula milk. Therefore, the longer breastfeeding duration in the present study should be evaluated within the context of the sample characteristics and limited sample size.

More than half of the participants (57.1%) reported using traditional aversive methods such as applying unpleasant substances to the nipple to end breastfeeding. The remaining 42.9% used verbal communication and reasoning with their infants. In a study by Oflu (2020), 85.9% of mothers used traditional methods, often learned from elder relatives. Similarly, a study from Konya, Turkey reported that 27.2% of mothers weaned suddenly, while 39.2% used traditional techniques (Oflu 2020). A qualitative study in Jordan found that confusing and conflicting advice from family and friends contributed to the preference for traditional methods (Altunel and Özaydın 2022; Abu Shosha 2022). The continued widespread use of traditional methods suggests that the breastfeeding cessation process is significantly shaped by the transmission of cultural knowledge and experience. Especially in societies with strong family ties, mothers often draw upon the experiences of their own mothers, mothers‐in‐law, or close relatives during this process (Aksoy et al. 2020; Oflu 2020; Alsaç and Polat 2018). The limited availability of professional guidance on breastfeeding cessation or insufficient focus on this period by healthcare services may make mothers more likely to resort to culturally transmitted practices. In this context, the breastfeeding cessation process can be considered not merely an individual choice, but a multifaceted experience shaped by social relationships, family dynamics, and cultural norms.

Participants cited several motivations for continued breastfeeding, including the infant's perceived need, enjoyment of the process, enhancement of maternal infant bonding, desire for equal treatment among children, and religious beliefs. In a qualitative study by Uçtu and Uludağ (2022), mothers described breastfeeding as a sacred and fulfilling experience (Uçtu and Uludağ 2022). Güleroğlu et al. (2023) also emphasized bonding, emotional security, and health benefits as key reasons for continued breastfeeding (Güleroğlu et al. 2023). The similarity of the reasons for continuing breastfeeding to previous studies suggests that this behavior is not merely an individual preference. The decision to breastfeed is often intertwined with societal expectations and cultural norms regarding the role of motherhood. The perception of breastfeeding as a sign of being a “good mother” can strengthen mothers' motivation to continue this process. In this context, breastfeeding practice can be linked to the socially shaped dimension of maternal identity and evaluated within a framework consistent with theoretical approaches to maternal role development.

Among the reasons for cessation, fatigue, emotional exhaustion, declining milk supply, maternal health conditions, pregnancy, and social pressure were frequently mentioned. Similar factors have been reported in other national and international studies (Güleroğlu et al. 2023; Kartal and Deniz‐Acar 2022; Ezenduka et al. 2018). “Breastfeeding aversion” or tolerance fatigue has been described as a major reason for maternal‐led breastfeeding cessation decisions (Bay 2022). The fact that fatigue, burnout, and the strain on personal boundaries were prominent reasons cited by participants for ending breastfeeding indicates that this decision cannot be explained solely by individual preference. The breastfeeding process is intertwined with the intense responsibilities and care expectations that come with the role of motherhood. Especially in situations where social support is limited, this process can lead to both physical and emotional strain on the mother. In this respect, the process can be evaluated as an experience with relational and social dimensions, not only individual but also in the context of maternal burden and emotional labor.

Cessation methods reported in the literature include gradual reduction, abrupt breastfeeding cessation, and aversive techniques such as applying unpleasant substances. While gradual and communicative methods are often preferred in clinical guidelines, aversive methods are still commonly used, possibly due to their perceived effectiveness and cultural transmission (Altunel and Özaydın 2022; Abu Shosha 2022). In our study, 8 of the 14 mothers used aversive or abrupt techniques. Studies have shown that these methods may lead to adverse emotional responses in infants, such as fear, rejection, or confusion (Kartal and Deniz‐Acar 2022; Aksoy et al. 2020).

Factors facilitating the breastfeeding cessation process included maternal determination, feeling prepared, having a supportive environment, infant cognitive readiness, and reduced suckling demand. On the contrary, the lack of social support, maternal emotional unpreparedness, desire to avoid upsetting the child, and the habit of breastfeeding to sleep were barriers to a smoother breastfeeding cessation process. In our sample, 9 out of 14 mothers reported receiving no professional or educational support. This supports previous findings that mothers frequently rely on family members or informal networks rather than healthcare providers (Altunel and Özaydın 2022; Oflu 2020; Abu Shosha 2022). The fact that mothers seek help from family members rather than healthcare professionals during the process of breastfeeding cessation should not be considered merely an individual choice. The fact that breastfeeding services largely focus on initiation and maintenance can lead to limited structured professional guidance for the breastfeeding cessation process. Furthermore, especially in societies with strong family ties, the experience of motherhood is often seen as a field of knowledge passed down through generations, and the experiences of close relatives are perceived as more accessible and reliable. Additionally, since breastfeeding cessation is often not perceived as a medical problem, the search for professional support can be limited. This situation demonstrates that the process is shaped by both cultural factors and the existing healthcare system.

Following cessation, many mothers experienced a sense of relief and emotional clarity. However, emotional discomfort such as sadness, guilt, regret, and anxiety was also reported. These emotions mirror symptoms of postpartum mood disturbances and may be associated with hormonal shifts following the reduction in oxytocin and prolactin levels (Güleroğlu et al. 2023; Abu Shosha 2022; Tadi 2019). Although the physiological changes associated with the end of lactation can affect mood, this experience is also intertwined with psychological and contextual factors. In particular, sudden or traditional methods applied without a child's preparation process of ending lactation, the lack of adequate professional guidance, and feelings of guilt or loss experienced by mothers can contribute to intensified emotional responses. Therefore, the cessation of breastfeeding can be considered more than just a biological process; it is a multifaceted transition related to maternal identity and attachment experiences. It is also conceivable that this transition could be experienced differently if appropriate support mechanisms were in place.

Infants also displayed a variety of behavioral responses. While some adapted easily, showing increased affection and improved appetite, others exhibited irritability, increased clinginess, or avoidance of the breast. These behavioral shifts may reflect developmental transitions, as well as the impact of the cessation method used. Infants subjected to abrupt or aversive methods were more likely to experience distress, as previously documented in related literature (Gürarslan Baş et al. 2018; Kartal and Deniz‐Acar 2022; Aksoy et al. 2020). The process of breastfeeding cessation should be considered not only a change in feeding but also a developmental transition. This period may coincide with a stage where the child's attachment patterns are reorganized, the separation process becomes more pronounced, and self‐regulation skills need support. Therefore, how the breastfeeding cessation process takes place is important. Traditional methods applied abruptly and without preparing the child can increase stress responses by challenging the child's sense of security; while gradual and sensitive approaches can facilitate the child's adaptation to this transition in a more harmonious way. In this context, breastfeeding cessation should be considered not only as a change in feeding but also as a process with relational and developmental dimensions.

## 6 Conclusion

This study explored the experiences of Turkish mothers during the process of breastfeeding cessation. The findings indicate that decisions to stop breastfeeding were mainly influenced by maternal fatigue, sleep deprivation, perceived decline in milk supply, pregnancy, and social pressures. Mothers often relied on traditional breastfeeding cessation practices due to limited access to professional guidance. While some mothers experienced relief and improved well‐being after cessation, others—particularly those who used aversive methods—reported feelings of regret, sadness, and concerns about their child's emotional adjustment.

Overall, the results highlight the need for greater professional involvement in guiding mothers through the breastfeeding cessation process. In line with international recommendations on respectful and supportive breastfeeding care, evidence‐based, gradual, and child‐centered strategies should be integrated into postpartum services to support maternal well‐being and minimize emotional distress for both mothers and infants during breastfeeding cessation. By conceptualizing breastfeeding cessation as a distinct and meaningful transition, this study contributes to the literature by addressing an underexplored phase of the breastfeeding journey and providing culturally grounded insights that may inform more sensitive and supportive maternal health practices.

## 7 Strengths and Limitations

A strength of this study is its qualitative design, which provided rich insight into the lived experiences of mothers and revealed cultural dimensions of breastfeeding cessation. However, the findings should be interpreted with caution due to several limitations. First, the sample size was relatively small (*n* = 14), which restricts the generalizability of the results. Second, the reliance on self‐reported narratives may introduce recall or social desirability bias. Third, because the study was conducted within the Turkish cultural context, the findings reflect culture‐specific practices and beliefs, which may limit transferability to other populations. One limitation of this study is the use of online recruitment, which may have resulted in a sample skewed toward mothers with higher educational levels and greater digital literacy. This may limit the transferability of the findings to mothers with limited access to digital platforms. Finally, although rigorous translation and coding procedures were applied, some nuances may have been lost in the interpretation process. Despite these limitations, the study contributes valuable knowledge to an underexplored area and can inform future research.

## 8 Implications for Practice: Recommendations for Midwives and Nurses

- Midwives and nurses should incorporate structured assessment of breastfeeding status and maternal intentions regarding breastfeeding cessation into routine postpartum and child health visits. When breastfeeding cessation is anticipated, professionals should provide evidence‐based guidance emphasizing gradual, responsive, and developmentally appropriate approaches.
- Clinical counseling should include exploration of maternal motivations for cessation, as understanding fatigue, return‐to‐work pressures, or perceived milk insufficiency may allow targeted interventions that support continued breastfeeding when desired.
- Midwives and nurses should be equipped to recognize factors that complicate breastfeeding cessation, such as infants' reliance on breastfeeding for sleep, emotional regulation, or closeness. Professional support may benefit from helping mothers explore alternative ways of maintaining closeness and soothing their infants during the breastfeeding cessation process.
- Screening for maternal exhaustion, sleep disruption, and emotional distress should be integrated into breastfeeding cessation‐related consultations. Providing both psychosocial support and practical management strategies may reduce premature or distress‐driven cessation.
- Educational programs for midwives and nurses should emphasize skills in communication and culturally sensitive counseling, enabling them to balance respect for traditional practices with the promotion of evidence‐based approaches.

## 9 Future Research

Further studies are needed to explore the long‐term psychosocial effects of different breastfeeding cessation methods on both mothers and children. Quantitative and mixed‐methods research with larger and more diverse samples could validate these findings and assess the effectiveness of professional counseling interventions designed to support mothers during the cessation process.

## Author Contributions

S.H. contributed to the literature review, the development of the conceptual framework, study design, data collection, data transcription, data coding, and drafting of the manuscript. A.S.K. critically reviewed the manuscript and provided intellectual input. N.A.B. contributed to data analysis, interpretation of the findings, and critical revision of the manuscript for important intellectual content. All authors read and approved the final version of the manuscript and agree to be accountable for all aspects of the work.

## Conflicts of Interest

The authors declare no conflicts of interest.

## Supporting information


**Data S1:** Semi‐structured interview guide.referes

## Data Availability

Data sharing not applicable to this article as no datasets were generated or analysed during the current study.
