# Supplementary material for: A Qualitative Study on Mothers' Experiences of Breastfeeding Cessation in Turkey
Source: Jpn J Nurs Sci. 2026 Jul 30;23(4):e70069. doi: 10.1111/jjns.70069 (PMC13423879; doi:10.1111/jjns.70069)
Supplement: Supplementary file 1 — Data S1: Semi‐structured interview guide.referes [file JJNS-23-e70069-s001.docx]

**Appendix 1. Semi-Structured Interview Guide**

1. **How would you describe your breastfeeding experience?**
   - How did it begin?
   - How did it continue over time?
2. **What are your thoughts and beliefs about breastfeeding?**
   - What do you know about breastfeeding? (e.g., whether it is necessary, its benefits)
3. **How did you feel during the breastfeeding period?**
   - Did you breastfeed willingly, with enjoyment, or out of obligation?
4. **Before you began the process of breastfeeding cessation, what changes did you notice in yourself and in your baby/child?**
   - Did you feel physically and psychologically ready?
   - Do you think your baby/child was ready?
5. **How did you end the breastfeeding process?**
   - How did you decide to stop breastfeeding?
   - Did people around you (e.g., your mother, spouse, or other family members) influence your decision regarding the timing or method?
   - How did you choose the method you used for breastfeeding cessation?
6. **What emotions did you experience during the breastfeeding cessation process?**
   - How did you feel the night before, during, and after stopping breastfeeding?
   - How did you feel in response to your baby’s reactions?
7. **How was your adjustment process after breastfeeding cessation, and how do you evaluate this experience?**
   - How was the adjustment process after cessation (your physical reactions, emotional responses), and how did your baby respond (e.g., sleep patterns, changes in feeding)? How long did this process last?
   - Did you have any regrets (e.g., wishing you had used a different method or questioning whether it was the right time)?
